# Rediscovery of Traditional Plant Medicine: An Underestimated Anticancer Drug of Chelerythrine

**DOI:** 10.3389/fphar.2022.906301

**Published:** 2022-06-01

**Authors:** Nianzhi Chen, Yulin Qi, Xiao Ma, Xiaolin Xiao, Qingsong Liu, Ting Xia, Juyi Xiang, Jinhao Zeng, Jianyuan Tang

**Affiliations:** ^1^ Department of Oncology, Hospital of Chengdu University of Traditional Chinese Medicine, Chengdu, China; ^2^ Hospital of Chengdu University of Traditional Chinese Medicine, Chengdu, China; ^3^ State Key Laboratory of Southwestern Chinese Medicine Resources, School of Pharmacy, Chengdu University of Traditional Chinese Medicine, Chengdu, China; ^4^ Hospital of Chengdu University of Traditional Chinese Medicine, School of Clinical Medicine, Chengdu University of Traditional Chinese Medicine, Chengdu, China; ^5^ Department of Gastroenterology, Hospital of Chengdu University of Traditional Chinese Medicine, Chengdu, China; ^6^ TCM Regulating Metabolic Diseases Key Laboratory of Sichuan Province, Hospital of Chengdu University of Traditional Chinese Medicine, Chengdu, China; ^7^ Geriatric Department, Hospital of Chengdu University of Traditional Chinese Medicine, Chengdu, China

**Keywords:** chelerythrine, traditional botanical drugs, anticancer, molecular mechanism, future direction

## Abstract

In many studies, the extensive and significant anticancer activity of chelerythrine (CHE) was identified, which is the primary natural active compound in four traditional botanical drugs and can be applied as a promising treatment in various solid tumors. So this review aimed to summarize the anticancer capacities and the antitumor mechanism of CHE. The literature searches revolving around CHE have been carried out on PubMed, Web of Science, ScienceDirect, and MEDLINE databases. Increasing evidence indicates that CHE, as a benzophenanthridine alkaloid, exhibits its excellent anticancer activity as CHE can intervene in tumor progression and inhibit tumor growth in multiple ways, such as induction of cancer cell apoptosis, cell cycle arrest, prevention of tumor invasion and metastasis, autophagy-mediated cell death, bind selectively to telomeric G-quadruplex and strongly inhibit the telomerase activity through G-quadruplex stabilization, reactive oxygen species (ROS), mitogen-activated protein kinase (MAPK), and PKC. The role of CHE against diverse types of cancers has been investigated in many studies and has been identified as the main antitumor drug candidate in drug discovery programs. The current complex data suggest the potential value in clinical application and the future direction of CHE as a therapeutic drug in cancer. Furthermore, the limitations and the present problems are also highlighted in this review. Despite the unclearly delineated molecular targets of CHE, extensive research in this area provided continuously fresh data exploitable in the clinic while addressing the present requirement for further studies such as toxicological studies, combination medication, and the development of novel chemical methods or biomaterials to extend the effects of CHE or the development of its derivatives and analogs, contributing to the effective transformation of this underestimated anticancer drug into clinical practice. We believe that this review can provide support for the clinical application of a new anticancer drug in the future.

## Graphical Abstract

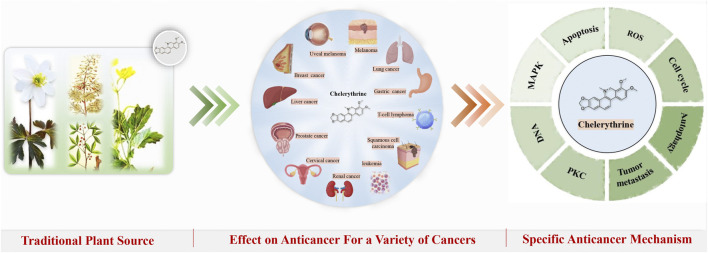

## 1 Introduction

Cancer is one of the primary causes of mortality worldwide and has killed nearly 9.6 million people in 2018 (Bray et al., 2018), leading to a heavy global economic burden and an impact on the quality of life. Over several decades, research on the diagnosis and treatment of cancer has rapidly developed, leading to significant advances in clinical practice, particularly in targeted cancer therapies and immunotherapy. However, cancer remains inevitable due to the increased probability of tumor recurrence, the lack of anticancer drugs, and the adverse effects and resistance to conventional treatments (Deng and Cassileth, 2013; Jermini et al., 2019).

Traditional herbal medicine is rich in bioactive ingredients and is widely used in clinical practice. Recently, herbal medicine extracts have played an increasingly important role in the discovery of available medicines for the prevention and treatment of cancer, with nearly 80% of the therapeutics for cancer therapy including natural products, derivatives/analogs, or imitation of natural products (Banik et al., 2019; Hsieh et al., 2015; Newman et al., 2003; Shanmugam et al., 2017; S. F.; Yang et al., 2013). Natural products (i.e., alkaloids, polyphenolic compounds, diterpenoids, flavonoids, and sesquiterpenes) present in various parts of plants possess immense anticancer potential (Bishayee and Sethi, 2016; Millimouno et al., 2014; S. F.; Yang et al., 2013). Among these natural products, alkaloids and polyphenols offer distinct advantages in cancer treatment (Newman and Cragg, 2016).

Based on a literature review, four herbaceous plants, *Chelidonium majus* L.*, Macleaya cordata* (Willd.) R. Br.*, Sanguinaria canadensis* L., and *Zanthoxylum asiaticum* (L.) Appelhans, Groppo & J.Wen*,* which are well known for their treatment of cancer, skin diseases, gastrointestinal parasites, liver cirrhosis, jaundice, and other difficult miscellaneous diseases, have a long history of usage, especially for treating cancer, possibly leading to a deep exploration of the effective anticancer active compounds based on the traditional use of these plants. Isoquinoline alkaloids are the common pharmacologically relevant substances of these plants (Croaker et al., 2016; Ni et al., 2016; Zielińska et al., 2018; Zeng et al., 2021). As a premier member, CHE is an alkaloid that is extracted mainly from the abovementioned four plants and possesses numerous biological properties, including anti-inflammatory (Niu et al., 2011), anticancer (Jana et al., 2017), antidiabetic (Zheng et al., 2015), antifungal (Wei et al., 2017; He et al., 2018), and anti-parasitic activities (Li et al., 2011), as well as the activity against ethanol-induced gastric ulcers (Li et al., 2014). Therefore, CHE can be used as one of the main representatives of the anticancer effect of these four traditional plants, combined with the needs of contemporary cancer drugs; it is worth further exploring the anticancer effect of CHE.

The antitumor activity of CHE has been demonstrated in *in vitro* experiments for cytotoxicity and growth delay against various tumor cell lines (Chmura et al., 2000). CHE induces apoptosis in cancer cells, suggesting that CHE is an anticancer agent (Malikova et al., 2006). A large number of studies have succeeded in identifying the anticancer activity of CHE in a variety of cancers, such as uveal melanoma (Kemény-Beke et al., 2006), leukemia (Vrba et al., 2008), non–small cell lung cancer (Gao et al., 2010), triple-negative breast cancer (Lin et al., 2017), prostate cancer (Malíková et al., 2006), liver cancer (Zhang et al., 2011), and renal cancer (Chen et al., 2016). The anticancer effect of CHE has been confirmed and has received considerable research attention; however, this underestimated anticancer drug is not used in clinical treatments, and it is worth discussing the reasons.

**FIGURE 1:**
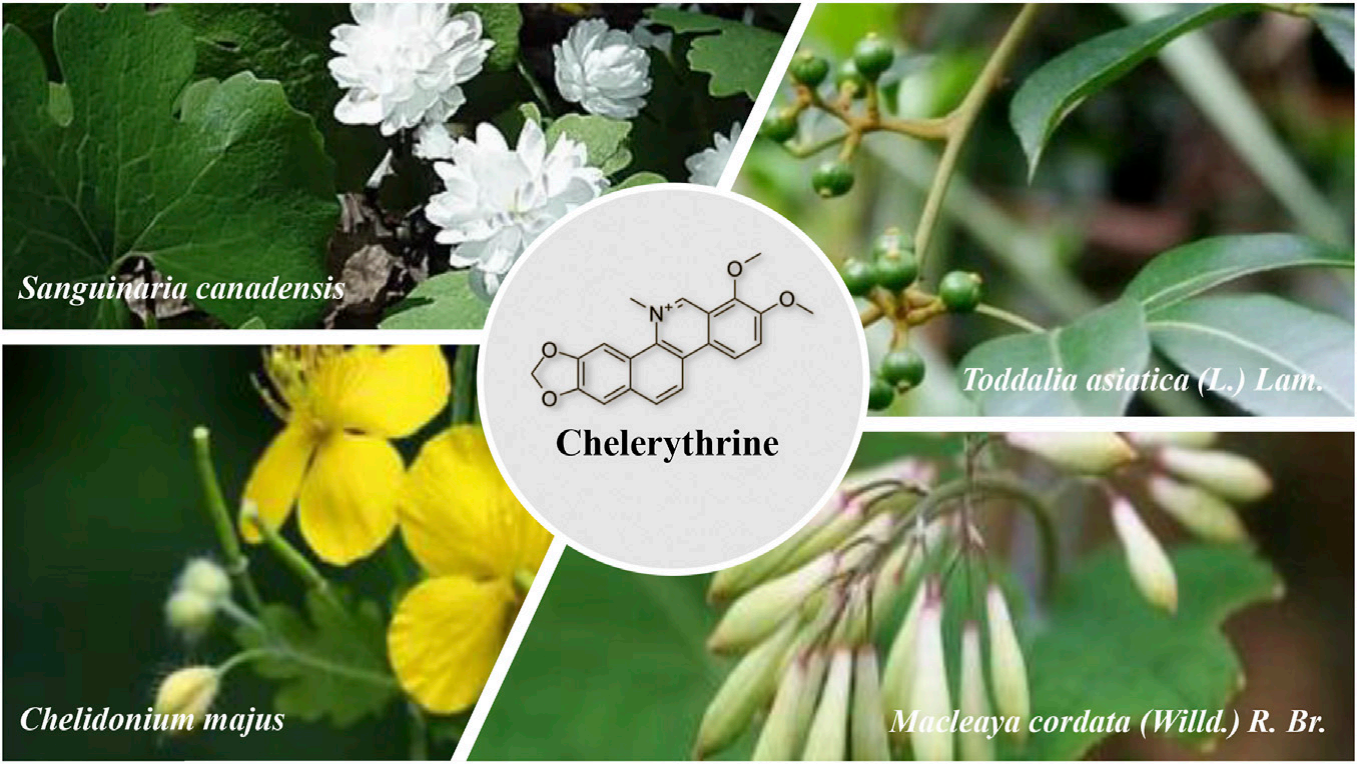
Plant sources of CHE.

Although the four plants and their related compounds have certain anticancer activities that have been reported, there is still a lack of systematic reviews of CHE. This review article aimed to investigate and systematically cover all the findings on the molecular mechanisms of CHE *in vitro* and *in vivo*, especially the cancer-combating properties. Further studies are needed to explore the clinical application prospects of CHE and provide systemic evidence for the anticancer activity. (Figure 1).

## 2 Plant Sources and Structural Features of Chelerythrine

### 2.1 Plant Sources of Chelerythrine and the History of Usage

**FIGURE 2:**
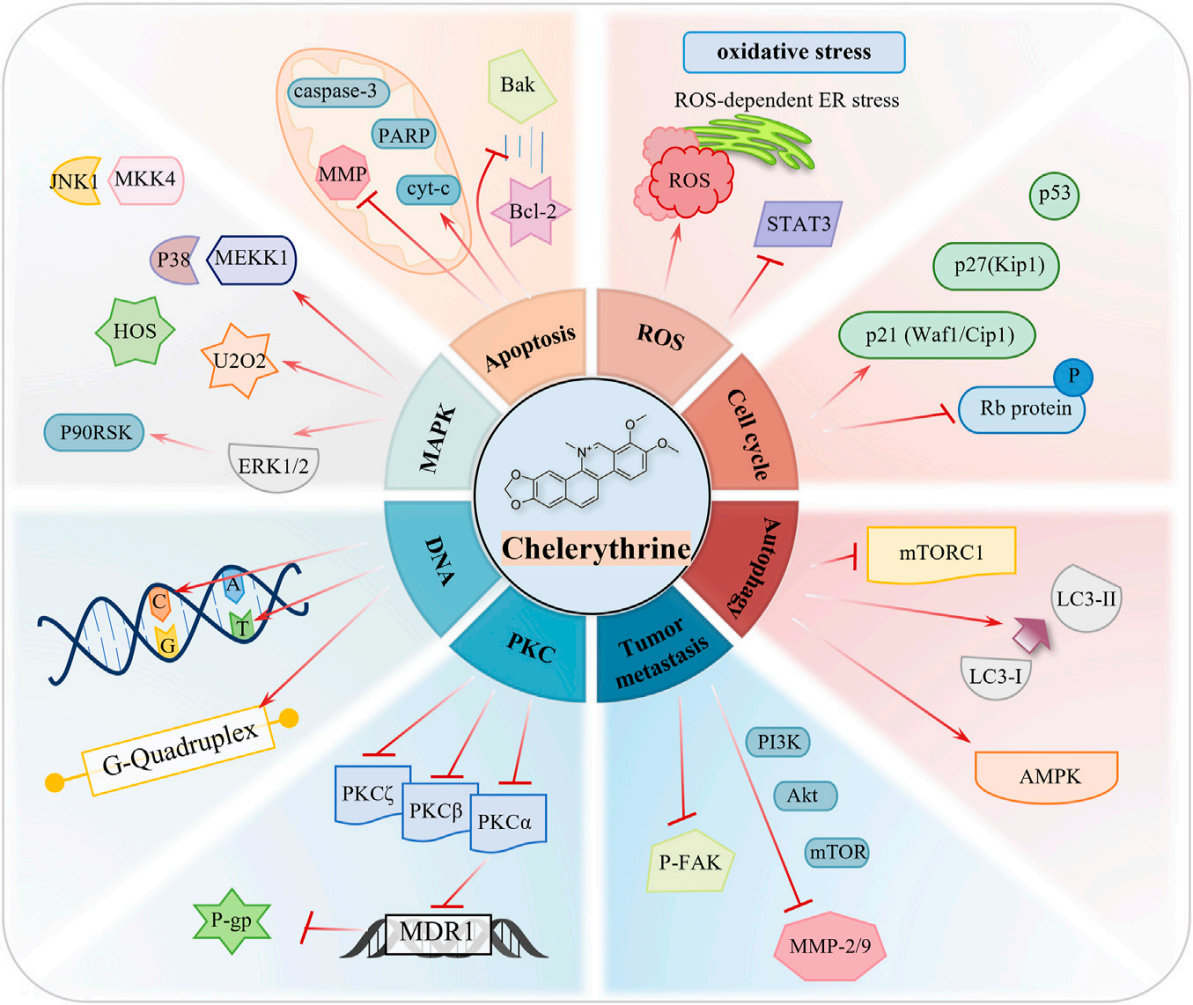
Chemical structure and anticancer strategy of CHE.

Chelerythrine (CHE) consists mainly of extracted and isolated alkaloid components from four plants of the families Papaveraceae (e.g., *Chelidonium majus* L., *Macleaya cordata* (Willd.) R.Br., and *Sanguinaria canadensis* L.) and Rutaceae (e.g., *Zanthoxylum asiaticum* (L.) Appelhans, Groppo & J.Wen) (Zhang et al., 2005; Malikova et al., 2006; Wang et al., 2013). Direct scientific studies have proven that the abovementioned four plants have antitumor medicinal values (Iwasaki et al., 2010; Deljanin et al., 2016; Ali et al., 2020; Tuzimski et al., 2021)**,** and CHE, as the most representative active compound for these plants, has also undergone a large number of studies on anticancer activity *in vitro* and *in vivo*, so there is a clear link between the compound and the traditional uses and the effects of these plants (Chmura et al., 2000). (Figure 2)

#### 2.1.1 Traditional Use of *Chelidonium. majus* L

CHE is one of the main effective compounds of the traditional herbal medicine *Chelidonium. majus* L. of the Papaveraceae family, which has a long history of clinical treatment. The plant *C. majus,* or greater celandine, is widely distributed in Asia, Europe, North Africa, and North America and is an admired plant with tremendous medicinal value that has been used in clinical treatment from antiquity (Gilca et al., 2010; Zielińska et al., 2018).

In folk medicine experience in Europe, *C. majus* is used to treat skin diseases in many areas, such as removing warts (Koleva et al., 2015), and *C. majus* has significant effects on skin wounds, scabies, ulcers, and skin eruptions (Mamedov et al., 2005; Jarić et al., 2007; Menkovi et al., 2011). The second widely applied medicinal value is for the treatment of liver diseases and jaundice, where *C. majus* can relieve precancerous lesions of liver cirrhosis and treat hepatitis and can be used for inflammation of the gallbladder and bile duct (Jarić et al., 2007; Savikin et al., 2013; Kujawska et al., 2016, 2017). Moreover, this herb has been used mostly to cure eye diseases since the Middle Ages, protecting against eye infections and sight impairment (Mayer et al., 2002; Kujawska et al., 2017). With the development of science, the medicinal value of this plant has been further explored, the anticancer activities of *C. majus* have also been investigated, and more verifiable scientific experiments have been conducted *in vitro* and *in vivo* in the traditional use of *C. majus*. In recent decades, numerous reports have proven the antitumor actions of *C. majus*, regardless of whether it is a plant extract of *C. majus* or a compound from this plant, manifesting efficacy in multiple cancer models with different molecular mechanisms (Biswas et al., 2008; Deljanin et al., 2016; Havelek et al., 2016; I et al., 2015; Park et al., 2015; Warowicka et al., 2019). In addition, several clinical trials have also demonstrated the beneficial effects of *C. majus* in the treatment of human cancer (Uglyanitsa et al., 2000; Gansauge et al., 2002; Ernst and Schmidt, 2005; Habermehl et al., 2006). More than 50 alkaloids have been isolated from this plant (Maji and Banerji, 2015), and the extracts and purified compounds exhibit a wide range of biological activity. The antitumor activity supports the clinical treatment of cancer, so the antitumor properties of this plant have been the primary focus (Mayer et al., 2002; Savikin et al., 2013). Major pharmacologically relevant components of *C. majus*, most of which are isoquinoline alkaloids, include berberine, chelerythrine, chelidonine, coptisine, and sanguinarine (Colombo and Bosisio, 1996). The antiproliferative, proapoptotic, and cytotoxic effects of berberine, chelerythrine, and sanguinarine on cancer cell lines of *C. majus* are based on their abilities (Zielińska et al., 2018). The anticancer activities of berberine and sanguinarine have been reviewed (Jin et al., 2016; Achkar et al., 2017), and it is necessary to dig deeper into CHE.

#### 2.1.2 Traditional Use of *Zanthoxylum asiaticum* (L.) Appelhans, Groppo & J.Wen

CHE is also one of the most characteristic compounds for *Zanthoxylum asiaticum* (L.) Appelhans, Groppo & J.Wen (another name is *Toddalia asiatica* (L.) Lam.), a plant from the family Rutaceae that is distributed mainly in tropical Africa and southeastern Asia and the southern areas of China (Xia and Liu, 2007), where the whole plant has been used as a drug for hundreds of years. *T. asiatica* has been chronicled as a treatment for colds, rheumatism, analgesia, injury, and hemoptysis in China (Shi et al., 2011), while the plant has also been used in the treatment of malaria in traditional practice in Africa, where the other most frequent diseases were followed by cough, chest pain, and sore throat (Ngarivhume et al., 2015; Orwa et al., 2013, Orwa et al., 2008). Based on these traditional medicinal experiences, further modern pharmacological studies have found antimalarial, antibacterial (Duraipandiyan and Ignacimuthu, 2009; Raj et al., 2012), and antidiabetic activities (Irudayaraj et al., 2013). At the same time, the antitumor effect of *T. asiatica* (Iwasaki et al., 2010; Li et al., 2018) and its drug activity in the treatment of arthritis and osteoporosis were also found (Watanabe et al., 2015; K.; Yang et al., 2013). Consequently, *T. asiatica* has often been used in folk medicine to treat different diseases, including but not limited to injuries, arthritis, and tumors.

According to the “Chinese Materia Medica” issued by the State Administration of Traditional Chinese Medicine, *T. asiatica* is used to treat malignant sores and lumps with the efficacy of detoxification, which is closely related to treating cancer. Based on the traditional use of this plant to treat cancer, multiple modern experimental studies have proven its anticancer activity(He and N, 1998; Iwasaki et al., 2010; Li et al., 2018) and anti-inflammatory activity (Kumagai et al., 2018; Ni et al., 2020). In recent years, with in-depth research, the chemical components in *T. asiatica* have gradually been discovered to be mainly coumarins and alkaloids, and alkaloids in *T. asiatica* play an important role in antitumor effects, especially benzophenanthridine alkaloids. CHE is the main benzophenanthridine alkaloid in this plant (Zeng et al., 2021). Combined with traditional experiences and studies, the anticancer activity of *T. asiatica* is worthy of in-depth exploration, and due to the important antitumor effect derived from CHE, in this article, the anticancer effect of CHE is comprehensively expounded in recent years to provide a reference for the related research and application of this medicinal material.

#### 2.1.3 Traditional Use of *Macleaya cordata* (Willd.) R. Br

*Macleaya cordata* (Willd.) R. Br. is an herbaceous plant that also belongs to the Papaveraceae family and is widely dispersed in China; the plant is called “Bo Luo Hui” and includes mainly alkaloids such as sanguinarine and CHE (Kosina et al., 2010). This traditional drug was used to relieve pain and has positive effects on the treatment of skin diseases, sores and ulcers, and rheumatism in folk experience (Lin et al., 2018). Modern studies have identified that the pharmacological actions of *M. cordata* can reduce inflammation and antibacterial and antiviral activities (Sedo et al., 2002; Ni et al., 2016), and *M. cordata* is extensively used in the treatment of skin diseases such as scabies and corporis (Luo, 1987), some gynecological diseases, pneumonia, bacterial infections(Wu and Zhu, 2002), expulsion of parasites, and cancer (Chen et al., 2020).

Meanwhile, similar to *T. asiatica,* according to the “Chinese Materia Medica” issued by the State Administration of Traditional Chinese Medicine, *M. cordata* was also effective in the treatment of malignant sores and lumps in traditional Chinese medicine. The current studies suggest that *M. cordata* has anticancer properties, and its purified compounds and/or crude extract possess antitumor activity (Lin et al., 2018). *M. cordata* extracts exhibited antitumor activity in cancer (Ali et al., 2020), and alkaloids extracted from *M. cordata* exhibit bioactivity and anticancer properties, as well as strongly inhibiting the proliferation of cancer cell lines, so these alkaloids are the main pharmacological evidence that *M. cordata* can be used to treat cancer (Liu et al., 2013; Si et al., 2020; Sai et al., 2021). Therefore, further pharmacological studies have revealed that alkaloids are the major bioactive components of *M. cordata* (Chen et al., 2009). To date, 147 alkaloids have been identified based on their chemical structures. Most of these compounds are isoquinoline alkaloids; furthermore, the two main components sanguinarine and chelerythrine are considered responsible for the pharmacological activity of *M. cordata* (Lin et al., 2018). Therefore, the anticancer effect of CHE is closely related to *M. cordata.*

#### 2.1.4 Traditional Use of *Sanguinaria canadensis* L

*Sanguinaria canadensis* L. is native to eastern North America. As a member of the Papaveraceae, *S. canadensis* contains six quaternary benzophenanthridine alkaloids (QBAs) (Bambagiotti-Alberti et al., 1991), while CHE is the second most important alkaloid in *S. canadensis*. *S. canadensis* is a traditional medicinal plant in Europe and North America that is used for treating pain, vomiting, gastrointestinal bleeding, abdominal mass, hemorrhagic tuberculosis, wound infection, gangrene, asthma, pertussis, pneumonia, diphtheria, dysentery, functional dyspepsia, jaundice, and liver disease (Croaker et al., 2016).

*S. canadensis* has a long history of curing cancer in clinical use since the mid-19th century, and people have used a black salve derived from *S. canadensis* (Jellinek and Maloney, 2005; Lin et al., 2018) to cure skin cancer (Croaker et al., 2016). Basic research also confirmed the possibility of its application in the treatment of human cancer through the *in vivo* experiments involving the *S. canadensis* extracts (Tuzimski et al., 2021). The anticancer activity of sanguinarine, the main component of *S. canadensis*, against various human malignancies has been validated and reviewed (Achkar et al., 2017). However, as the second major component of *S. canadensis*, CHE also has a clear and broad anticancer effect and can also represent the traditional anticancer use of *S. canadensis,* which can be explored deeply.

Almost all four of these plants have the same natural active compounds and traditional uses with analgesic, antibacterial, anti-inflammatory*,* and detoxification functions, and the most important frequently used traditional applicatio*n* is the anticancer effect. Among these compounds, isoquinoline alkaloids are the main pharmacological components that are well known for their anticancer effect. The most important alkaloids found in these plants, such as sanguinarine and CHE, can represent traditional anticancer uses and have a clear and more pharmacologically evidence-based demonstration of anticancer potential, providing new anticancer strategies for clinical practice (Achkar et al., 2017). In recent years, an increasing number of studies have identified that CHE has a significant broad-spectrum anticancer effect but is underestimated. Many direct scientific studies and references have proven that CHE can be used as the most important pharmacological component of the abovementioned four plants for anticancer purposes. Therefore, based on the efficacy of these traditional plants, this article further summarizes the anticancer effect of CHE and identifies new strategies for the treatment of cancer.

### 2.2 Structural Features of Chelerythrine

CHE (C_21_H_18_NO_4_) is a natural benzophenanthridine alkaloid with the chemical name of 1,2-dimethoxy-12-methyl-[1,3]-benzodioxolo-[5,6-c] phenanthridinium, and the relative molar mass is 348.37. According to the pH range, CHE exists in two forms, “charged iminium” in the pH range 1–6 and “alkanolamine” form in the pH range 8.5–11, which transform into each other in an equilibrium reaction and have extensive biological and biochemical performance *in vivo.* The studies reveal that the iminium form of CHE has a high binding affinity to DNA, but the neutral alkanolamine form does not bind to DNA. However, in the context of cancer, the intra-tumoral pH is low due to the acidic environment, and the functionality of this compound can be better. (Basu et al., 2013). CHE interacts with a variety of proteins and DNA, and the charged iminium form binds more strongly to DNA/RNA, while the alkanolamine state has more binding affinity to bovine serum albumin (Bhuiya et al., 2016). The most active part of CHE is the carbon adjacent to the quaternary nitrogen, attacked by the nucleophiles, and responsible for this compound’s observed toxicity.

## 3 AntiCancer Strategy of Chelerythrine

**FIGURE 3:**
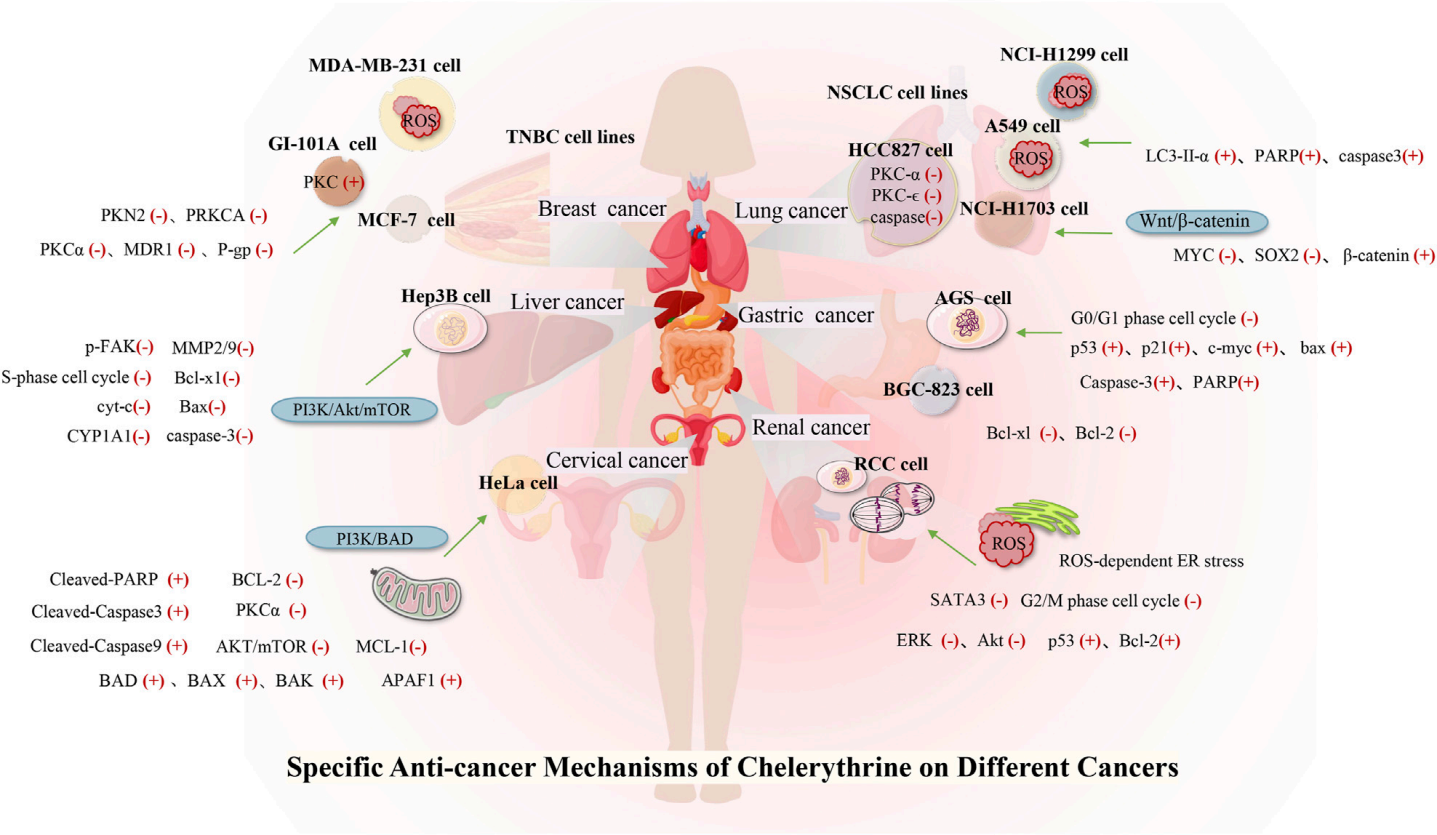
Role of CHE in a variety of human cancers.

The anticancer targets and mechanisms of natural products have raised greater concern and have been widely reported throughout the years (Ren et al., 2019). Evidence based on multiple cancer experiments indicates that CHE is a broad-spectrum anticancer drug, which can be a prospective compound in the treatment of cancers. CHE causes the death of cancer cells through cell apoptosis, cell cycle arrest, autophagy, inhibition of invasion and metastasis, and generation of reactive oxygen species (ROS). (Figure 3; Table 1).

**TABLE 1:** Tabular representation of the reported functions.

Mechanism	Phenotype of anticancer effects
Caspase effect mechanism (cleaved-PARP↑, caspase-3↓, cleaved-caspase-3↑, Bax↑, Bcl-xL↓, Bcl-2↓, caspase-9↓, cleaved-caspase-9↑, cle-caspase-8↑, and cyt-c↑)	Cancer cell viability↓, cancer cell death, or apoptosis↑
MMP-2↓, MMP-9↓, and p-FAK↓	Cell migration and invasion↓
p53↑, c-Myc↓, and PI3k/AKT (Akt↓, p-Akt↓, and p-PI3Ks↓)	As a cancer suppressor gene, the p53 protein regulates the mechanism of apoptosis inhibition. As an oncogene, the activation of c-Myc causes excessive proliferation of cells. The PI3k/AKT signaling pathway plays an important role in apoptosis.
MAPK (p-MKK4↑,p-MKK3↑, p-JNKs↑, p-MEK↑, p-p38↑, and p-ERK1/2↑)	The ERK pathway is the most prominent and clinically utilized target, while the JNK pathway and p38 pathway play critical modulatory roles in cancer cells.
Autophagy (LC3-I↓, LC3-II↑, ROS↑, and mTOR↓)	Autophagy is a form of programmed cell death, and inducing autophagy may be an essential strategy against cancer.
PKC (PKC-α↓ and PKC-ϵ↓)	PKC can affect division and proliferation by catalyzing some small-molecule peptides and enzymes that bind to DNA in tumor cells.
ROS-mediated apoptosis (ER stress↑, ROS↑, p-STAT3↓, ATF4↑, and H_2_O_2_↑)	High levels of ROS can activate a variety of proapoptotic signaling pathways.
G-quadruplex	G-quadruplex has become one of the critical targets for developing antitumor drugs, and CHE has good selectivity for G-quadruplex while interacting with DNA
p21↑ and p27↑	Cell cycle arrest.
Mitochondria (mitochondrial membrane potential↓ and cyt-c↑, ROS↑)	The mitochondrial membrane potential is decreased, the permeability of the mitochondrial membrane is increased, and the proapoptotic factors are released into the cytoplasm.

### 3.1 Inducing Cancer Cell Apoptosis

The human body eliminates damaged and aged cells to maintain homeostasis through a genetic apoptosis process under physiological conditions, while tumor cells evade apoptosis and grow indefinitely. Cells perform apoptosis programs through external or internal pathways. The external pathway is composed of ligands and death receptors that bind to Fas-related death domains (FADDs) and tumor necrosis factor receptor 1 (TNFR1)-related death domain (TRADD) interactions, which then form the death-inducing signal complex (DISC), assembly, activate caspase-8, and further initiate cell apoptosis (Wong, 2011). The intrinsic mitochondrial pathway is regulated by the Bcl-2 family. Bax and Bcl-2 bind to the mitochondrial membrane and contain proapoptotic or antiapoptotic proteins. Apoptotic bodies are formed by recruiting Apaf-1 and procaspase-9 to the cytoplasm, further triggering the downstream caspase-9/3 cascade reaction (Wong, 2011).

The anticancer ability of CHE is focused on cell apoptosis. Micromolar concentrations of CHE have been reported to significantly inhibit tumor cell growth, and this inhibitory effect is related mainly to the induction of apoptosis. In a radioresistant tumor model, CHE caused apoptotic cancer cell death by increasing sphingomyelinase activity and enhancing ionizing radiation (IR)–mediated cell killing (Chmura et al., 1997). Studies have found that CHE is capable of aggregating in mitochondria, which could cause mitochondrial dysfunction because of its cellular permeability and mitochondria-targeting ability (Lei et al., 2018). CHE has been used as an inhibitor of Bcl-2 family proteins (Herbert et al., 1990) and could significantly inhibit cell proliferation in tumor cells, inducing cell apoptosis by adjusting the expression of Bcl-2 family proteins and activating the pathway of mitochondrial apoptosis (Zhang et al., 2011). Chan et al. (2003) screened 12 species extracted from 107,423 natural products that can inhibit the binding of Bcl-xL and Baks, in which the four active ingredients were CHE. Therefore, further research confirmed that CHE is a functional inhibitor of Bcl-xL, bound at the BH region of Bcl-xL (Zhang et al., 2006), and was reported to interfere specifically with the interaction between Bcl-xL and Bax, which act directly on the mitochondria from tumor cells and lead to the release of Cyt-c. CHE treatment induces cancer cell apoptosis by activating caspase-3 and poly-ADP-ribose polymerase (PARP) and reducing Bcl-2 and mitochondrial membrane potential (MMP) (Zhang et al., 2012).

### 3.2 Inducing Reactive Oxygen Species-–Mediated Apoptosis

ROS are a typical byproduct of various cellular processes. As an important second messenger of signals, ROS can regulate cell death and cell proliferation (Covarrubias et al., 2008). High levels of ROS can activate a variety of proapoptotic signaling pathways, and cellular antioxidative machinery can balance the damaging effect of excessive ROS (Milkovic et al., 2019). Cancer cells typically obtain higher levels of ROS than normal cells to resist persistent internal oxidative stress (Cairns et al., 2011). Hence, targeting ROS and oxidative stress can be considered an excellent method to combat cancer cells.

Indeed, several studies have demonstrated that CHE induces ROS-mediated apoptosis in several types of cancer (Tang et al., 2017; Wu et al., 2018; He et al., 2019). CHE treatment can increase ROS generation in cancer cells, exceeding the internal antioxidant processing capacity and inducing cell death. A high level of ROS is also related to DNA and protein damage, which in turn, is related to the occurrence of apoptosis (Pelicano et al., 2004). In addition, CHE can mediate tumor cell death through ROS-dependent endoplasmic reticulum (ER) stress and STAT3 inactivation (Wu et al., 2018; He et al., 2019). Studies on five types of tumor cells, MCF-7, MDA-MB231, and HCT116, and one type of nontumor cell, AA-8, by Matkar et al. found that CHE induced a significant generation of ROS through the redox reaction cycle, especially hydrogen peroxide H_2_O_2_, which mediates rapid cell apoptosis (Matkar et al., 2008). The redox reaction cycle also consumes a large number of reducing coenzymes, which further accelerates cell apoptosis. Tumor cell necrosis and apoptosis were observed after treatment with CHE (Kemény-Beke et al., 2006; Vrba et al., 2008). Higher concentrations of ROS have a strong cytotoxic effect on cells, which indicates that CHE can promote not only cell apoptosis but also cell necrosis. CHE was shown to mediate the two-way death of tumor cells that produce a large amount of ROS in cells, which is one of the ways that CHE exerts antitumor activity.

### 3.3 Effect of Chelerythrine on Cell Cycle Arrest

Several cyclin-dependent kinases regulate the cell cycle that controls cell division and proliferation. Therefore, regulating cell cycle checkpoints that induce cell cycle arrest is a therapeutic target for cancer treatment (Otto and Sicinski, 2017). CHE was reported to have potent activity in cell cycle arrest, imparting anticancer effects on the human promyelocytic leukemia cell line by accumulating at the G1 phase and increasing at the G2/M phase when treated with a higher concentration (Vrba et al., 2008). Furthermore, when treating prostate cancer cell lines, CHE has a significant cytotoxic effect and induces cell cycle arrest by increasing cyclin kinase inhibitors p21^Waf1/Cip1^ and p27^Kip1^ (Malíková et al., 2006). CHE can also induce S-phase cell cycle arrest to inhibit cancer cell proliferation in gastric cancer (Zhang et al., 2012).

### 3.4 Inhibition of Tumor Invasion and Metastasis

Tumor metastasis is one of the causes of death in most cancer patients, especially due to the migration and invasion of cancer cells from the primary part and subsequent infiltration into foreign tissues (Valastyan and Weinberg, 2011). Therefore, blocking the metastatic tumor process with natural products would be efficient in preventing cancer metastasis. The expression and activation of the matrix metalloproteinases MMP-2 and MMP-9 are only increased in breast cancer patients (Scorilas et al., 2001), while MMP-2 induces cancer migration (Xu et al., 2005). CHE was found to downregulate the expressions of MMP-2 and MMP-9 through the PI3K/Akt/mTOR signaling pathway and change the cytoskeleton structure by reducing p-FAK, which inhibited the metastasis and invasion of cancer in a dose-dependent manner (Zhu et al., 2018).

### 3.5 Chelerythrine as an Effective Protein Kinase C Antagonist

Protein kinase C (PKC), which belongs to the serine/threonine kinase family and has multiple isoenzyme subtypes, was first discovered in the mouse brain in 1977 (Takai et al., 1977). PKC, closely related to the development of cancers and overexpressed in various tumor cells, can affect division and proliferation by catalyzing some small-molecule peptides and enzymes that bind to DNA in tumor cells.

CHE (IC_50_ = 0.66 μmol/L), a PKC inhibitor, not only binds to the catalytic domain and inhibits the expression of PKC (Herbert et al., 1990) but also affects the function of PKC by inhibiting the movement of PKCα, PKCβ, and PKCζ in a concentration-dependent manner(Chao et al., 1998; Siomboing et al., 2001). As a subtype of the PKC family, PKCα affects tumor growth, the cell cycle, cell metastasis, and cell apoptosis (Leitges, 2007; Konopatskaya and Poole, 2010), which regulates cancer cells through the phosphorylation of P-gp (Zhang et al., 2003). CHE has been proven to be a highly effective and selective inhibitor of PKCα (Melling et al., 2009), inducing cell apoptosis by inhibiting PKCα, downregulating the transcription of MDR1, and reducing the expression of P-gp, which also reversed multidrug resistance (Cao et al., 2011). Furthermore, CHE, in addition to PKC inhibition, has a non-competitive inhibitory action on the human P2X7 receptor itself, which needs further research in the future (Shemon et al., 2004).

### 3.6 Inducing Autophagy-Mediated Cell Death

Autophagy is a form of programmed cell death (Tsujimoto and Shimizu, 2005) that involves many diseases and physiological processes, including cancer and ageing (Levine and Kroemer, 2008; Yang et al., 2011). Autophagy can be induced by ROS production, hypoxia, nutritional deficiencies, and viral infections (Azad et al., 2009). Therefore, inducing autophagy may be an essential strategy against cancer.

mTOR is a central regulator of autophagy and is related to cell proliferation and regulated by adenosine monophosphate-activated protein (AMP)–activated kinase (AMPK), which overactivates cancer cells. Medvetz et al. (2014) screened 6,700 compounds against mTOR and found that CHE had the most substantial killing effect on mTORC1-overactive cells, inducing autophagy by inhibiting mTORC1 and increasing AMPK (Kim et al., 2011), which forms autophagosomes and converts LC3I to LC3II (Tanida et al., 2008; Mah and Ryan, 2011; Si et al., 2020). Likewise, CHE has been shown to enhance the expression of LC3-II in NSCLC A549 and NCI-H1299 cells (Tang et al., 2017). In addition, ROS can trigger prodeath or prosurvival autophagy in cancer cells (Chen et al., 2008). CHE-induced ROS-mediated autophagy was found to contribute to apoptosis in NCI-H1299 cells, while CHE-induced autophagy through the production of ROS is a concomitant effect in A549 cells.

### 3.7 Chelerythrine as a Selective Telomeric G- Quadruplex DNA Stabilizer and Telomerase Inhibitor

DNA has attracted attention as a drug target or potential antitumor target. Some small molecules that can bind to DNA, such as cisplatin, have become clinically applied antitumor drugs. The pharmacological activity of CHE, especially its anticancer activity, is closely related to the structural characteristics of its molecules, which readily bind to the base pairs in the DNA structure. *In vitro* studies experimenting with CHE treatment on mouse spleen cells and lymphoid leukemia L1210 cells found that the drug has a damaging effect on DNA in a dose-dependent manner (Kaminskyy et al., 2008; Urbanová et al., 2009). Three DNA adducts were detected after incubation with CHE and calf thymus deoxyribonucleic acid (CT-DNA) in the presence of Sprague–Dawley rat liver microsomes and nicotinamide adenine dinucleotide phosphate (NADPH) (Stiborová et al., 2002). Subsequently, CHE was shown to interact with DNA, according to multiple experiments (Bai et al., 2008, Bai et al., 2006, Bai et al., 2014). CHE could bind with DNA [*Ka*=(1.04 ± 0.11)×106], where the particular binding site of CHE is a continuous GC pairing sequence (5′-TGG​GGA-3′/3′-ACC​CCT-5′), and CHE is reported to have a specific combination character with single nucleotide bulge DNA with C and T. CHE interacts with CT-DNA mainly through the interlayer, which shows its therapeutic prospects with the ability to insert the interlayer and stabilize the DNA double helix structure (Li et al., 2012).

Moreover, G-quadruplex has become one of the critical targets for developing antitumor drugs, and CHE binds selectively to the telomeric sequence G-quadruplex (Bai et al., 2014). DNA and RNA can fold into a variety of alternative conformations. A particular nucleic acid structure called G-quadruplex plays an important role in malignant transformation and cancer development (Kosiol et al., 2021). As a promising target for cancer therapy, molecular and cell biology have demonstrated the G-quadruplex formation with key biological processes ranging from genome instability and cancer (Varshney et al., 2020). CHE exerts antitumor activity by possessing a solid affinity for inhibiting the telomerase activity by substrate sequestration through G-quadruplex stabilization, which leads to fast proliferation of cells, thus promoting tumor cell death (Noureini et al., 2017). The binding selectivity of CHE toward G-quadruplexes is confirmed by both experimental and theoretical determination (Terenzi et al., 2019). In addition, CHE is reported to bind to non-telomeric G-quadruplex sequences also in the promoters of other genes, which binds to G-quadruplexes at promoters of VEGFA, BCL2, and KRAS genes and downregulates their expression (Jana et al., 2017). CHE binds to the ds poly (rA) by the mechanism of intercalation, not only interacting with DNA but also interacting with double-stranded RNA, which may also be one of the anticancer mechanisms (Pradhan et al., 2017).

### 3.8 Regulation of the MAPK Signaling Pathway

Mitogen-activated protein kinase (MAPK) pathways are viable targets that have been widely studied for cancer therapy. The extracellular signaling–regulated kinase (ERK) pathway is the most prominent and clinically utilized target, while the Jun N-terminal kinase (JNK) pathway and p38 pathway play critical modulatory roles in cancer cells. These pathways can regulate metabolism and epigenetics and change the response of cancer cells to both targeted therapies and chemotherapies (Lee et al., 2020).

CHE was reported to preferentially activate the JNK1 and P38 MAPK pathways in cervical cancer cells (HeLa), inducing cell apoptosis *via* the oxidative stress-related MEKK1, MKK4-dependent JNK1, and P38 pathways (Yu et al., 2000). CHE-induced cell apoptosis is mediated by activating the MKK4/JNK1 and MEKK1/P38 pathways in osteosarcoma cell lines. Likewise, CHE was further reported to activate the MEK/ERK1/2 pathway in a concentration–time-dependent manner, stimulating the ERK MAPK cascade to induce apoptosis, while P90RSK was found to be a downstream kinase of ERK1/2. The activation of ERK and cell apoptosis induced by CHE could be significantly reversed by MEK1-specific inhibitors (UO126 and PD98059) (Yang et al., 2008).

## 4 Role of Chelerythrine in a Variety of Human Cancers

**FIGURE 4:**
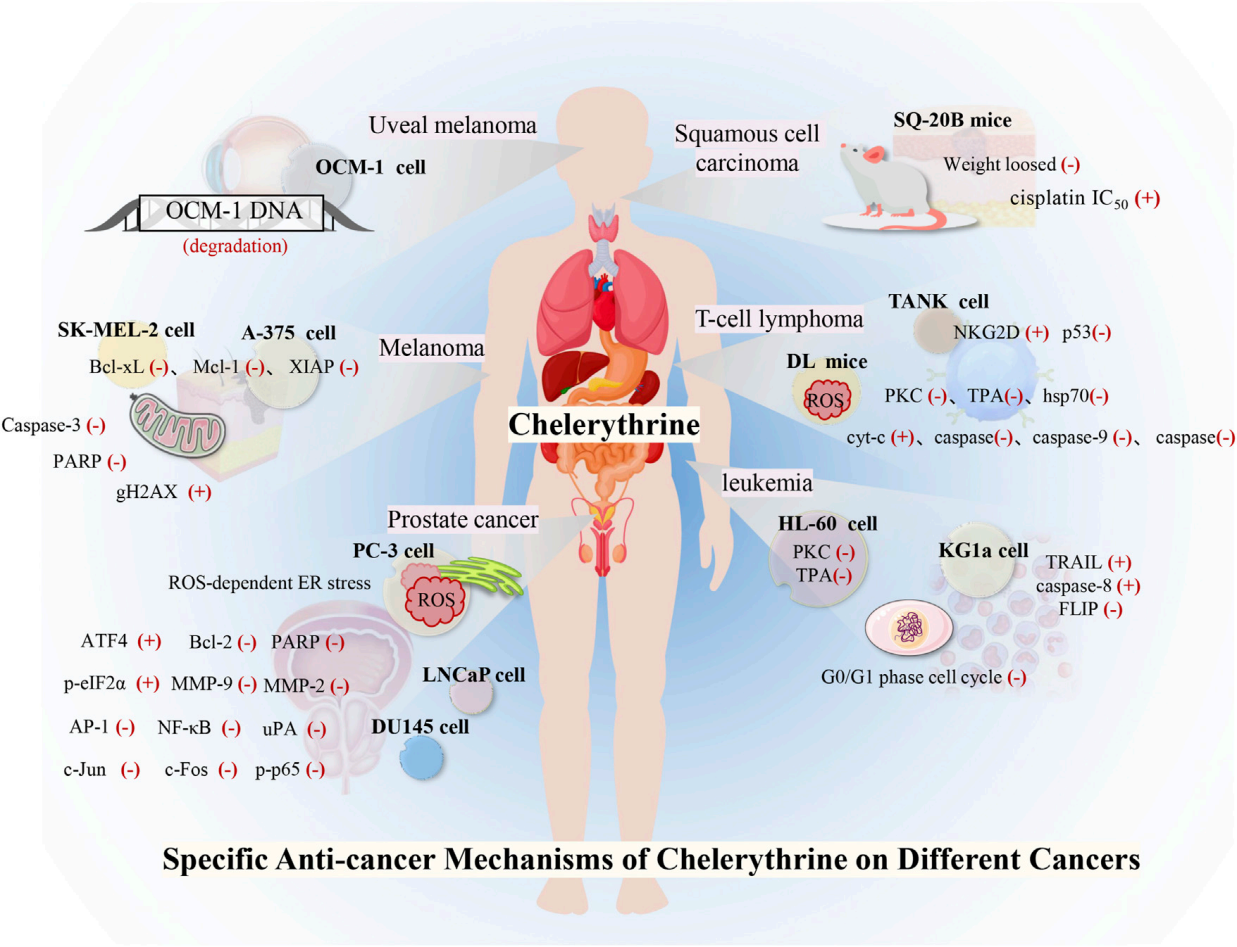
Role of CHE in a variety of human cancers.

**FIGURE 5:**
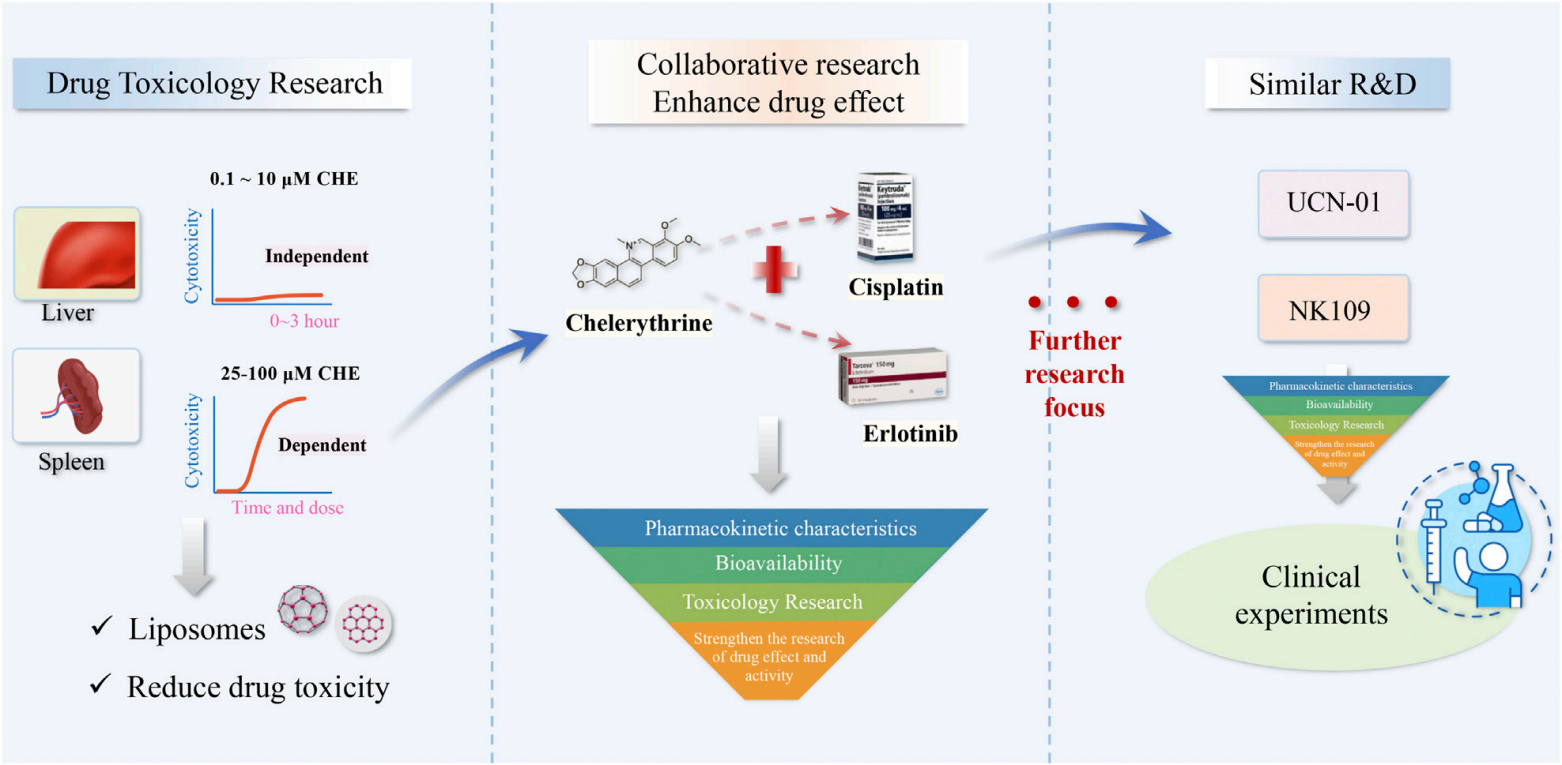
Toxicity and security of CHE and future direction.

Many studies have investigated the role of CHE against different types of cancer. Drug discovery programs have identified CHE as the main antitumor drug candidate. CHE is a multitargeted agent with broad-spectrum antitumor effects that can be used as a wonder drug for treating cancers (Table 2). Some of the studies evaluating the antitumor potential of this drug are summarized as follows (Figures 4, 5).

**TABLE 2:** Current evidence on anticancer effects of CHE

Cancer type	Cell line	Duration/dosage	Mechanism	Reference
Lung cancer	NCI-H1299 and A549 cells	10, 15, and 20 μM	Cell viability↓, cell death and apoptosis↑, LC3-I↓, LC3-II↑, cle-PARP↑, and cle-caspase-3↑	Tang et al. (2017)
	NSCLC cell lines (H1299, H460, A549, and cisplatin-resistant A549)	10 µM	Cell apoptosis↑, PKC-α mRNA, and protein↓	Gao et al. (2007)
	SK-LU-1 and human lung cancer stem cells (HLCSCs)	1.5, 3, 6.25, 12.5, 25, and 50 μg/ml	Cell migration and invasion↓, β-catenin↓, ROS↑, MYC↓, SOX2↓, and HLCS activity↓	Heng and Cheah, (2020)
	HCC827	0, 5, 10, 15, 20, 30, and 40 μM	ROS↑, PKC-ϵ↓, caspase-3↓, cell apoptosis↑, cell viability↓, and cell proliferation↓	Wang et al. (2021)
Liver cancer	SMMC-7721 cell	1.25, 2.5, 5, and 10 μg/ml	Cell proliferation↓, cell S-phase arrest↑, Bax↑, Bcl-xl↓, cell apoptosis↑, cyt-c↑, cle-caspase-3↑, and cle-PARP↑	Zhang et al. (2011)
	HCC	1.25, 2.5, and 5 μM	Cell proliferation↓, cell apoptosis↑, p-FAK↓, MMP-2/9↓, and cell metastasis and invasion↓	Zhu et al. (2018)
	HepG2	0.01, 0.1, and 1 μM	CYP1A1↓ and dioxin-induced 7-ethyxoresorufin-O-deethylase (EROD) activity↓	Zdarilová et al. (2006)
Gastric cancer	BGC-823 cells		Δψm↓, cyt-c↑, cle-caspase-3↑, cle-PARP↑, Bcl-xl↓, Bcl-2↓, and cell apoptosis↑	Zhang et al. (2012)
	AGS cells	1 μM, 10 μM, and 100 nM	Cell growth↓, Cells arrested at the G0/G1 phase↑, p53↑, p21(waf/cip1) ↑, c-Myc↓, bax ↑, and cell apoptosis↑	Zhu et al. (1999)
Breast cancer	MDA-MB-231, BT-549, HCC 1937, and MDA-MB-468	5 μM	Cell proliferation↓, cell cycle arrest↑, cell apoptosis↑, and chemotherapy activity↑	Lin et al. (2017)
	MCF-7 and MDA-MB-231	7.5 and10 μM	Cell viability↓, H_2_O_2_↑, ROS↑, p-H2AX↑, PARP cleavage↑, and cell apoptosis↑	Matkar et al. (2008); Xie et al. (2015)
	MCF-7 and MCF-7^Taxol^		PKCa↓, MDR1 gene↓, and P-glycoprotein (P-gp) ↓	Cao et al. (2011)
	GI-101A	50 μM	Cell growth↓ and PKC activity↑,	Fernandez et al. (2000)
	MCF-7	10^−8^ Μ	Cell proliferation↓ and calcium-dependent PKCs↑	Mueller et al. (1997)
Renal cancer	HEK-293 and SW-839 cells	5 and 10 μM	Cell growth↓, p-ERK↓, p-Akt↓, p53↑, Bcl-2-associated X protein↑, Bcl-2↓, caspase-3↓, PARP↓, and tumor growth↓	Chen et al. (2016)
	Caki and 786-O	6, 9, and 12 μM	Cell viability↓, G2/M cell cycle arrest↑, ROS-dependent ER stress↑, p-STAT3↓, and cell apoptosis↑	He et al. (2019)
Prostate cancer	LNCaP and DU145 cells	0.1, 0.5, 1, 5, and 10 μmol/L	Cell proliferation↓, p21Waf1/Cip1 ↑, p27^Kip1^↑, p16^Ink4^↓, retinoblastoma protein↓, and cell apoptosis↑	Malíková et al. (2006)
	PC-3 cells	10 μM	Bcl-2↓, cle-PARP↑, H_2_O_2_↑, ROS↑, ER stress↑, p-eIF2α↑, and ATF4↑	Wu et al. (2018)
	DU145 and PC-3 cells	5 and 10 μM	Cell proliferation↓, cell migration and invasion↓, MMP-2↓, MMP-9↓, uPA↓, TIMP-1↑, and TIMP-2↑, PAI-1↑, PAI-2↑, NF-κB↓, AP-1↓, p-p65↓, c-Fos↓, and c-Jun ↓	Yang et al. (2020)
Cervical cancer	HeLa cells	0, 2, 4, and 6 μM,	BAD↑, BAX↑, BAK↑, BCL-2↓, MCL-1↓, cell apoptosis↑, cell proliferation↓, p-PI3Ks↓, AKT↓, mTOR↓, and PKCα↓,	Yang et al. (2020)
Uveal melanoma	OCM-1	8 μg/ml	DNA degradation↑, cell apoptosis↑, and necrotic cell death↑	Kemény-Beke et al. (2006)
Melanoma	A-375, SK-MEL-2, and A-375-p53DD	0.1, 0.5, 1, 1.5, 2, and 3 mg/ml	Cell proliferation↓, cell apoptosis↑, Bcl-xL↓, Mcl-1↓, XIAP↓, caspase-3↓, and PARP↓	Hammerová et al. (2011).
	A375, G-361, SK-MEL-3		Cell viability↑ and cytotoxic activity↑	(Tomasz et al., 2021)
Dalton’s lymphoma	DL cells	10 μg/ml	Cell vitality and proliferation↓, cell apoptosis↑, HSF1↓, and hsp70↓	Kumar et al. (2013)
	DL cells	1–30μM	PKC↓,cyt-c↑,Apaf-1↑,caspase-9↑,caspase-3↑, HSF1 phosphorylation↓, and cell apoptosis↑	Kumar and Acharya, (2014)
	DL-bearing BALB/c (H^2d^) mice	2.5 mg/kg	Survival duration↑, cytotoxic function↑, and recovery immunosuppression↑	Kumar et al. (2015a)
	DL cells	10 μM	Total-p53/p-p53(ser-15) ↑, cyt-c↑, cle-caspase-9↑, cle-caspase-3↑, and degradation of DNA↑	Kumar et al. (2015b)
Leukemia	HL-60 cells	0.5 μM	Cell differentiation↓	Zheng et al. (2005)
	HL-60 cells	0, 1, 1.5, 2, and 5 μM	Cell cytotoxicity↑, cell viability↓, cell cycle arrest in G1 phase↑, cell cycle distribution↑, cell apoptosis and necrosis↑, cle-caspase-9↑, and cle-caspase-3↑	Vrba et al. (2008)
	KG1a	1, 2, 3, 4, and 5 mol/L	TRAIL-induced apoptosis↑, cle-caspase-8↑, and FLIP↓	Platzbecker et al. (2003)
	CEM T-leukemia human cells	8 μg/ml	EMC cells↑, cell apoptosis↑, fragmentation of DNA↑, and enzyme PARP-1↑	Kaminskyy et al. (2008)
	Mouse lymphocytic leukemia cells, L1210	0.5–8 mg/ml	Cell cytotoxicity↑, plasma membrane integrity↓, DNA damage↑, and cell apoptosis↑	Kaminskyy et al. (2008)
Squamous cell carcinoma	SQ-20B MCF7 breast (wt p53; Ref. 38) and MCF7ADR breast (resistant to adriamycin),	10 μm; 2.5 and 5 mg/kg	Cell apoptosis↑, tumor growth↓, and weight loss↓	Chmura et al. (2000)
	UM-SCC, 8029NA, and 8029DDP	10 μM	Cisplatin IC_50_ values↓ and cisplatin sensitivity ↑	Hoffmann et al. (2002)

### 4.1 Lung Cancer

Lung cancer is the most common malignant tumor, with 1.38 million deaths occurring each year. In lung cancers, approximately 80–85% are non–small cell lung cancer (NSCLC), and 10–15% are small cell lung cancer (Wan et al., 2018). Despite the continuous improvement of detection and therapeutic methods, NSCLC is still difficult to diagnose and has a poor prognosis. Reports stated that CHE efficiently enhanced the drug sensitivity of NSCLC cell lines (including cisplatin-resistant A549) to cisplatin by inhibiting the expression of PKC-α and elevating tumor cell apoptosis rates (Gao et al., 2007). CHE induced cell apoptosis in lung cancer cell lines (A549 and NCI-H1299) by suppressing the protein expression of LC3-II and increasing the protein expressions of PARP and cleaved caspase-3 (Tang et al., 2017). A549 cells have a higher antioxidant ability than NCI-H1299 cells (Sun et al., 2016), making it much easier to generate ROS in lung cell lines, which results in autophagic cell death (Martin and Barrett, 2002). CHE can affect ROS-mediated autophagy-induced cell death in lung cancer cell lines (Tang et al., 2017). Another study on the NSCLC cell line (HCC827) reported the efficacy of CHE in inducing apoptosis and inhibiting cell growth by inducing ROS release and downregulating caspase-3 and PKC-ϵ expression. CHE imparted anticancer effects on HCC827 by inhibiting PKC and caspase-3 activation, which were blocked by PMA, a PKC activator, thereby proving that CHE inhibited cancer cell viability and proliferation and induced cell apoptosis through the ROS/PKC-ϵ/caspase three pathway and glycolysis (Wang et al., 2021).

In addition, an *in vivo* study proved that CHE exerted specific anticancer effects on concentration adjustment to inhibit the Wnt/β-catenin pathway in different lung cancers and facilitate cancer cell apoptosis, inhibiting the proliferation and diminishing the self-renewal capacity of human lung cancer stem cells (HLCSCs) by downregulating β-catenin, SOX2, and MYC (Heng and Cheah, 2020; Wang et al., 2021). Owing to the inhibition of the tumorigenic activity of HLCSCs by CHE, which can inhibit cancer growth, the CHE concentration can be flexibly adjusted to protect against different specific targets in different cancers. A novel CHE analog, namely, compound 3f, was also found to induce dose-dependent G0/G1 cell cycle arrest, which enhanced selectivity against NSCLC tumor cells (Yang et al., 2016).

### 4.2 Liver Cancer

Most liver cancer patients have a definitive diagnosis and reach an advanced stage due to early unobvious symptoms. Currently, the small application range of targeted therapy drugs and recurrence and drug resistance after radiotherapy and ablation treatment lead to the unoptimistic overall treatment effect of advanced liver cancer, and the 5-year survival rate is low (Schlachterman et al., 2015).

CHE was reported to inhibit the proliferation of HCC cells and promote cell apoptosis, inhibiting metastasis and invasion of HCC in a dose-dependent manner by downregulating the expression of MMP-2/9 through the PI3K/Akt/mTOR pathway (Zhu et al., 2018). CHE not only significantly inhibits liver cancer cell proliferation through cell S-phase arrest, regulating the protein Bcl-2, increasing the proapoptotic protein Bax, and downregulating Bcl-xl but also disrupts the mitochondrial membrane potential. Activated caspase-3 and cleaved PARP result in apoptosis in a dose-dependent manner (Zhang et al., 2011). Moreover, some studies have pointed out that the biological effects of CHE may be related to the aryl hydrocarbon receptor (AhR). Dvořáka et al. (2006) found that CHE did not activate AhR in the rat hepatoma cell line H4IIE at any time or dose test and had no effect on the transcription of AhR. CHE exerted biological effects by inhibiting the catalytic activity of CYP1A1 in the human liver cancer cell line HepG2 but did not affect the expression of CYP1A1 (Zdarilová et al., 2006).

### 4.3 Gastric Cancer

Gastric cancer is the second leading cause of cancer-related deaths, owing to its high mortality (Lee et al., 2014). Gastric cancer has a higher level of PKC activity than normal tissues. As a PKC inhibitor, CHE can suppress cell growth and induce apoptosis in a gastric cancer cell line (AGS) by inducing G0/G1 phase arrest, as evaluated by the expressions of p53, p21 (waf/cip1), c-myc, and Bax (Zhu et al., 1999). The compound was also found to arrest the S phase, decrease Δψm, and release Cyt-c from mitochondria into the cytoplasm in a dose-dependent manner, which subsequently activated caspase-3 to cleave the PARP protein. CHE also imparted apoptotic effects on the human gastric cancer cell line BGC-823 by downregulating the expressions of Bcl-xl and Bcl-2 (Zhang et al., 2012).

### 4.4 Breast Cancer

Breast cancer has become the leading cause of female cancer death, and existing breast cancer chemotherapy drugs have limitations such as large adverse reactions, easy drug resistance, and lack of selectivity (DeSantis et al., 2019). Triple-negative breast cancer (TNBC) currently lacks therapy. One of the potential targets of TNBC is PKC, and PKN2 is highly expressed in TNBC. CHE showed potent anticancer activity against TNBC that overexpressed PKN2 and PRKCA by inhibiting cell growth and inducing apoptosis (Lin et al., 2017), selectively inhibiting growth with a half-inhibitory concentration (IC_50_) of 1.6 μM for MDA-MB-231 cells and increasing ROS generation, thereby inducing apoptosis (Matkar et al., 2008; Lin et al., 2017).

CHE (50 μM) can block the cell growth and activation of PKC induced by HCQ and prednisone treatment in GI-101A breast cancer cells (Fernandez et al., 2000). Selective stimulation of calcium-dependent PKCs has been reported to block the proliferation of MCF-7 cells (Mueller et al., 1997). CHE inhibited the PKC activity in MCF-7 and MCF-7^Taxol^ cells, downregulated the level of MDR1, and reduced the level of P-gp (Cao et al., 2011). CHE, effectively extracting (UHPE) and isolating (PZRCCC), was more active against breast cancer cells than the positive control (vincristine = 5.04 μg/ml) (Ali et al., 2020).

### 4.5 Renal Cancer

Renal cancer accounts for 2%–4% of all adult malignant diseases worldwide, with a mortality rate of more than 40% (Znaor et al., 2015). Renal cell carcinoma (RCC) accounts for approximately 70% of renal cancer cases (Novick, 2007), and approximately half of patients are resistant to chemotherapy and radiotherapy (Znaor et al., 2015). CHE selectively killed human RCC cells *via* inhibition of cell viability and induction of G2/M phase arrest (Caki and 786-O), also showing proapoptotic properties in RCC through the activation of ROS-dependent ER stress and the inhibition of STAT3 phosphorylation (He et al., 2019). Another study on human renal cancer cell lines (SW-839) showed that CHE could inhibit the proliferation and growth of renal cancer cells in a time- and dose-dependent manner by decreasing the phosphorylation of ERK and Akt, upregulating p53 and Bcl-2, and cleaving caspase-3 and PARP protein. CHE effectively reduced tumor growth in a xenograft tumor model, perhaps indicating that CHE affects renal cancer *via* inhibition of the ERK activity (Chen et al., 2016).

### 4.6 Cervical Cancer

Cervical cancer is the most common female cancer worldwide. The potency of CHE against cervical cancer cells (HeLa) was evaluated by Yang et al. in 2020. They reported that CHE inhibited cell proliferation by triggering mitochondrial apoptosis through the PI3K/BAD signaling pathway, significantly increasing the activated expression of the BAD protein, upregulating the proapoptotic proteins BAX and BAK, and downregulating the antiapoptotic proteins BCL-2 and MCL-1 (Yang et al., 2020). Moreover, another study on HeLa cells reported the efficacy of CHE in inducing apoptosis by preventing the phosphorylation of PI3K and the suppression of AKT/mTOR and PKCα. CHE also triggered the mitochondrial “intrinsic” pathway by upregulating the expression of APAF1, cleaved-caspase3,9, and cleaved-PARP in HeLa cells (T. Yang et al., 2020).

### 4.7 Uveal Melanoma

Uveal melanoma, an intraocular malignant tumor that originates from melanocytes in the eyes, is an aggressive malignancy and easily metastasizes to other organs. CHE showed potent anticancer activity against uveal melanoma by catalyzing DNA fragmentation and apoptosis. CHE was reported to significantly induce DNA degradation and cell apoptosis of the human vein melanoma cell line OCM-1 and cause necrotic cell death in a dose-dependent manner (Kemény-Beke et al., 2006).

### 4.8 Head and Neck Squamous Cell Carcinoma

Head and neck squamous cell carcinoma (HNSCC) is a malignant tumor of squamous cell differentiation, and the incidence of HNSCC continues to rise (Ferlay et al., 2019; Johnson et al., 2020). Studies on HNSCC cell lines showed that CHE induced rapid radiation and chemotherapy-resistant cell apoptosis *in vitro*, while CHE significantly inhibited tumor growth in HNSCC tumor model mice with xenotransplantation, with a weight loss of less than 10% (Chmura et al., 2000). CHE interacted with PKC and significantly decreased cisplatin IC_50_ values in six cell lines derived from HNSCC in combination with cisplatin (IC_50_ = 0.4–5.8 μg/ml), which also increased the cisplatin sensitivity of the HNSCC cell lines (Hoffmann et al., 2002).

### 4.9 Melanoma

Although comprising only approximately 1% of skin cancers, melanoma is the most dangerous skin cancer with a high mortality rate, and the incidence has increased steadily (Dzwierzynski, 2013). *Sanguinaria canadensis* has been recorded to treat melanoma, and the main component, benzophenanthridine alkaloids, is a drug that can inhibit the growth and viability of melanoma cells through a variety of molecular mechanisms. Among these mechanisms, the anticancer potential of CHE was evaluated in human melanoma A-375 and SK-MEL-2 cell lines, where the drug was found to facilitate cell apoptosis by suppressing the expressions of antiapoptotic proteins (Bcl-xL, Mcl-1, and XIAP), decreasing mitochondrial membrane potential, and cleaving caspase-3 and PARP. The phosphorylation of histone H2AX was detected to evaluate the DNA damage related to behavioral activation therapy (BA) treatment, and CHE was further reported to increase the expression of gH2AX and regulate caspase-3 cleavage at different levels (Hammerová et al., 2011). CHE showed strong cytotoxic activity against human melanoma cell lines (A375, G-361, and SK-MEL-3) with IC_50_ values below 0.55 μg/ml compared with anticancer drugs (etoposide, cisplatin, and hydroxyurea), indicating that CHE has high anticancer potential in melanoma (Tuzimski et al., 2021).

### 4.10 T-Cell Lymphoma

T-cell lymphoma is characterized by highly deleterious, invasive, and rapid growth due to its unique etiology. CHE was reported not only to reduce the vitality of murine T-cell lymphoma (DL) cells but also to induce cell apoptosis. CHE also blocked the PKC activation that inhibited heat shock factor 1 (HSF1) and hsp70 expression (Kumar et al., 2013). Another study demonstrated that CHE induced cell apoptosis and increased ROS generation by inducing the permeability and rupture of the mitochondrial membrane, thereby inducing apoptosis of DL cells by releasing cyt-c and activating Apaf-1 and the cleavage of caspase-9,3 (Kumar and Acharya, 2014).

In addition, CHE significantly increased the protein and mRNA expressions of total p53/phospho-p53, which led to the induction of apoptosis by the p53 pathway, which was further confirmed by the p53 knockdown of DL cells (Kumar et al., 2015b). *In vitro*, CHE was also found to significantly delay the progressive growth of DL and increase the survival period of DL-bearing BALB/c (H^2d^) mice. The *in vivo* administration of CHE (2.5 mg/kg) results in increased cytotoxic function of TANK cells and recovery of immunosuppression caused by tumor invasion (Kumar et al., 2015a).

### 4.11 Leukemia

Leukemia is a type of malignant tumor affecting the hematopoietic system. PKC is involved in the pharmacological activity of 12-O-tetradecanoylphorbol-13-acetate (TPA)–induced growth inhibition and apoptosis in myeloid leukemia. As a broad inhibitor of the PKC family, CHE significantly decreased cell differentiation in the human promyelocytic leukemia (HL-60) cell line, which is induced by the combination of TPA and capsaicin (Zheng et al., 2005). A study on HL-60 cells stated that CHE exhibited stronger antiproliferative activity than other alkaloids (Hatae et al., 2015). CHE was reported to have a cytotoxic effect on HL-60 cells, which results in decreased cell viability in a dose-dependent manner and accumulation of the cell cycle at the G1 phase. CHE also induced apoptosis of HL-60 cells by activating mitochondrial apoptotic pathways (Vrba et al., 2008).

Recent data have shown that CHE promotes the FAS-mediated cell state in the immature (CD34+/38-) myeloid cell line KG1a. CHE induced KG1a cells to be sensitive to tumor necrosis factor-related apoptosis-inducing ligand (TRAIL), which activated caspase-8 and downregulated FLIP long and short (Platzbecker et al., 2003). Another study demonstrated the typical characteristics of apoptosis in CEM T-leukemia human cells treated with CHE through the changes in the microstructure of EMC cells, which led to the further development of apoptosis, including fragmentation of DNA and reparation enzyme PARP-1 (Kaminskyy et al., 2008). CHE was also reported to induce DNA damage, cell cytotoxicity, and disruption of plasma membrane integrity in L1210 leukemic cells, which were more sensitive to the toxic effect of CHE than normal mouse spleen cells. These results suggested that CHE possesses slightly selective toxicity against cancer cells without affecting normal cells (Kaminskyy et al., 2008).

### 4.12 Prostate Cancer

Prostate cancer is the most common malignancy in men worldwide (Prashanth, 2019). Advanced localized, recurrent, and metastatic diseases often portend poor outcomes (La et al., 2019). The endoplasmic reticulum (ER) plays an important role in the apoptosis signaling pathway (Ryu et al., 2017), and ROS levels are related to the ER stress pathway. CHE treatment was found to exhibit excellent anticancer effects in prostate cancer cells by inducing ROS-dependent ER stress (Wu et al., 2018), which reduced Bcl-2 and increased cleaved PARP. ROS accumulation in PC-3 prostate cancer cells *via* increased intracellular H_2_O_2_ levels was achieved by CHE. In addition, CHE induced ER stress by increasing the levels of p-eIF2α and ATF4 in a time-dependent manner (Ryu et al., 2017).

CHE was reported to inhibit cell growth and regulate the expression of cell cycle- and apoptosis-related proteins in human prostate cancer cell lines (LNCaP and DU145) (Malíková et al., 2006). CHE was further found to have a significant effect on inhibiting the invasion and metastasis of these androgen-independent cancer cells at concentrations of 5–10 μM. The compound decreased the MMP-9, MMP-2, and uPA proteins and augmented the expressions of PAI-1 and PAI-2. Meanwhile, CHE also suppressed NF-κB and AP-1, which was demonstrated by the decreased expressions of p-p65, c-Jun protein expression, and c-Fos (Yang et al., 2020).

## 5 Synergistic Interactions of Chelerythrine With Other Agents

Although the anticancer activity of CHE has been confirmed, CHE has not been widely used in human clinical trials. Therefore, researchers need to look for new CHE values from other perspectives. Many natural products have synergistic effects with other listed anticancer drugs (Li et al., 2017). The main advantage of combined therapy is reducing the dose and toxicity of chemotherapy while maintaining or even increasing its efficacy. Several studies have explored the advantages of combining natural compounds with classical chemotherapeutic drugs (Cai et al., 2002; Efferth et al., 2002).

CHE was found to enhance the inhibition of cell proliferation of the chemotherapy agent taxol on TNBC cells. Compared with the single-drug treatment, the combination treatment of dual drugs significantly reduced cell proliferation, indicating a promising therapeutic schedule for TNBC. The combination treatment of CHE with taxol had a synergistic effect with combination index (CI) values at ED50 (0.75190–1.13763) (Lin et al., 2017).

Combination treatment of CHE with cisplatin significantly decreased cisplatin IC_50_ values and increased cisplatin sensitivity in HNSCC cell lines, and the IC_50_ value of CHE was between 8.5 μM and 13.6 μM. CHE (IC50 = 10 μM) combined with increasing doses of cisplatin demonstrated an additive effect compared with the theoretical additive dose-response curves. However, the single use of CHE was always more effective in inducing cancer cell apoptosis than the combined treatment with cisplatin (Hoffmann et al., 2002).

The potentiated inhibitory effect of CHE combined with erlotinib was also evident against NSCLC cell lines, which resulted in a significant decrease in malignant cell features. The combination treatment effectively delayed tumor growth and blocked EGFR by decreasing the phosphorylation of STAT3, p38 MAPK, ERK1/2, and BAD proteins, while the IC_50_ value of CHE was between HCC827 (IC_50=_5.0 ± 0.48 μM), SK-MES-1 (IC_50=_6.35 ± 1.26 μM), and A459 (IC_50=_7.78 ± 0.56 μM) (He et al., 2017). In SK-MES-1 and A459 cells, which are less sensitive to erlotinib, CHE showed potentiated inhibitory effects compared with erlotinib. Similarly, growth inhibition and apoptosis induction in tumor cells showed significant synergistic antitumor effects in NSCLC cells when CHE was combined with cisplatin. CHE (72.4%) also had a better effect on inhibiting the growth of tumors than the single use of cisplatin (64.3%) (Gao et al., 2007).

These studies provide a new perspective on the anticancer therapeutic strategy of CHE in combination therapy. At present, studies on the synergy of CHE with other agents are limited to the abovementioned limited cancers. For CHE as a broad-spectrum anticancer drug, more options can be explored in the future.

## 6 Toxicity Study and Clinical Application of Chelerythrine

### 6.1 Toxicity and Security of Chelerythrine

CHE has abundant pharmacological activity in the treatment of cancers but has been greatly restricted in clinical use because of its toxic side effects and other shortcomings. Despite extensive investigations on the role and use of CHE in the prevention and treatment of cancers, only a few studies have mentioned its toxicity.

We also collected the toxicity reports of CHE from the literature, which provided useful data for further research and development of new drugs containing CHE. In the works of Huang et al., high-performance liquid chromatography–tandem mass spectrometry (HPLC–MS/MS) was used to detect the distribution of CHE *in vivo* and demonstrated that the concentrations of CHE in the liver after 48 h and spleen after 24 h were 20.64 ng/ml and 44.04 ng/ml, respectively, higher than those of other tissues (<15ng/g). The results also indicated that the metabolic enzymes of XO, NQO1, NQO2, and carbonyl reductase are involved in CHE reduction (Huang et al., 2021). Another study demonstrated that CHE triggered acute hepatotoxicity at a dose of 10 mg/kg/day, and any liver damage was not observed with the administration of 0.2 mg/kg intraperitoneally (i.p.) over 56 days (Ulrichová et al., 1996). Furthermore, they also demonstrated the nontoxic nature of CHE in human vs. porcine liver cells at dosages of approximately 0.1 and 10 μM in 0–3 h of incubation, while CHE showed dose–time-dependent toxicity within the range of 25–100 μM and mitochondrial dehydrogenase activity and the cellular glutathione levels suggesting that mitochondria are unlikely to be a primary target for CHE in the cell (Ulrichová et al., 2001). In addition, the destruction of cellular calcium homeostasis by CHE was observed with a notable Ca^2+^-ATPase (SERCA1)-inhibitory activity, suggesting a possible cause of the CHE cytotoxicity (Vieira et al., 2015).

Another experiment investigated the most toxic *Chelidonium majus* alkaloids (CALs) in rat hepatocytes and found that CHE (EC20 ≤ 2 μM) was one of the most toxic compounds, indicating a marked structure–toxicity relationship in hepatocyte cells (Gao et al., 2019). Studies have also indicated that CHE was safe under an average daily oral dose of 5 mg/kg, which did not cause any genotoxicity or hepatotoxicity (Kosina et al., 2003). However, different doses of CHE were administered to experimental Wistar rats, and the results showed that CHE increased cumulative mortality and had long-term toxicity on lung tissues in a dose-dependent manner. CHE can aggravate toxic lung damage at high dosages (>=5.6 mg/kg), possibly via NF-κB activation and the production of inflammatory cytokines (Liu et al., 2017). These studies have revealed that the toxicity of CHE may be related to the dosage and emphasized that a thorough investigation of long-term medication is needed.

Hence, controlling and reducing drug toxicity is the key aspect for CHE to enter human clinical research. Current toxicological studies provide us with data on safe doses of CHE. Recent studies provide some new materials that can reduce the toxicity of CHE. Pharmacokinetic studies have revealed that CHE long circulation liposomes (CHELPs) modified with polyethylene glycol remained stable and exhibited sustained drug release while increasing the bioavailability and alleviating the toxicity of CHE, which showed a good therapeutic effect against cancer (Li et al., 2013; Wang et al., 2021). This study showed that nanostructured material may be an effective method to reduce chelerythrine toxicity and may contribute to the development of novel drugs in clinical trials.

### 6.2 Pharmacokinetics of Chelerythrine

In recent years, there have been many studies on the pharmacokinetics of CHE in animals, but there is still a lack of human research. Some studies were conducted to investigate the single- and multiple-dose pharmacokinetics of chelerythrine (CHE) and its metabolite in animals. The AUC value for CHE was 0.52 ± 0.09 (ng/ml hr), and the calibration curves were linear over the concentration range of 0.5–100.0 ng/g for CHE in broiler chickens (Xie et al., 2015; Hu et al., 2019). After oral and IM administrations in pigs, CHE rapidly reached *C*_*max*_ of 5.04 ± 1.00 ng/ml at 1.83 ± 0.26 h and *C*_*max*_ of 69.79 ± 15.41 ng/ml at 0.42 ± 0.13 h, respectively. The half-life (*T*_1/2_) was 2.03 ± 0.26 h for CHE (Zhao et al., 2021). The results of these studies revealed that CHE is metabolized rapidly. At the same time, pharmacokinetic studies were also carried out from the extracts of these traditional plants, such as *Toddalia asiatica* (Qiu et al., 2022), *Chelidonium majus* L.(Zhou et al., 2013), and *Macleaya cordata* extracts (Shen et al., 2022). In pharmacokinetic studies of CHE in rat plasma through UPLC-Q-TOF-MS and UPLC-MS/MS after the oral administration, the results showed the plasma drug concentration of CHE was very low (Lin et al., 2020). Recent studies proved that the biomaterial might be a suitable method to enhance the bioavailability and improve the therapeutic efficacy of CHE *in vivo*. The relative bioavailability of CHE in liposomes (CHELPs) was significantly increased, and CHELP has a lower clearance rate and higher absorption concentration than the free drug (Li et al., 2013b; Wang et al., 2021).

### 6.3 Clinical Application

In fact, clinical evidence remains scarce, which is typical for many natural products isolated from medicinal plants, and CHE is no exception. At present, UCN-01 and NK109, CHE analogs, have entered the clinical trial stage as antitumor drugs (Gojo et al., 2013; Ma et al., 2013), and most of these drugs are used in combination with classical chemotherapeutic drugs. Among them, many pieces of evidence of efficacy suggest that UCN-01 has a notable curative effect in combination with classical chemotherapeutic drugs in patients with solid tumors (Sausville et al., 2001; Kortmansky et al., 2005; Hotte et al., 2006). Nonetheless, CHE is effective against a vast range of cancers and is expected to be used as a preparation for the clinical treatment of cancer, which needs further development based on pharmacological activity research. Further optimization of its structure and development of related analogs are also feasible methods. Studies related to the pharmacokinetic properties, toxicity, and bioavailability of CHE are of utmost importance and can provide a scientific theoretical basis for its clinical development and utilization, which has certain practical significance.

The toxicity studies, combination therapy, and clinical application of CHE indicate the focus of future research.

## 7 Conclusion

In conclusion, similar to artemisinin and paclitaxel, the experience of traditional Chinese medicine and herbs has provided us with a large number of abundant effective compounds, and further research and optimized composition will contribute to human health. Extensive efforts have been devoted to identifying the bioactivity and mechanism of CHE and publishing its effectiveness in the treatment of different cancers. CHE was reported to act upon numerous molecular targets and mechanisms that are the key roles associated with pathogenesis in cancers. Therefore, CHE has shown immense potential in the treatment of cancers. The antitumor activity of CHE is the comprehensive effect of proliferation, invasiveness, and apoptosis of tumor cells, which inhibits tumor growth through multiple molecular pathways with few side effects due to its proapoptotic potential and makes CHE a suitable candidate for the treatment of cancers.

CHE has shown immense potential in the treatment of cancers; however, CHE has not been widely used in human clinical trials due to its shortcomings, such as drug toxicity. Therefore, researchers need to look for new values from other perspectives. In addition, as an underestimated anticancer drug, more studies should broaden the scope of traditional uses and provide better preparations, particularly binding to different categories of active molecules, which requires more extensive investigation and interdisciplinary efforts. At present, studies on the synergy of CHE with other agents identified that combined therapy is reducing the dose and toxicity while maintaining or even increasing its efficacy, and nanostructured material may be an effective method to reduce CHE toxicity and increase the bioavailability. Further in-depth pharmacokinetic studies on humans and toxicology research, the mechanism of combination therapy, and research on new CHE-based biomaterials for reducing drug toxicity and structurally optimized analogs are required to enhance the efficacy and safety of CHE, and more clinical studies are required in the future to validate preclinical studies of CHE as an antitumor strategy.

This review summarized the anticancer abilities and the antitumor mechanism of CHE. It also highlighted the limitations and the current problems, and we believe that this review can provide support for the clinical application of a new anticancer drug in the future.

## Abbreviations

AMPK, adenosine monophosphate-activated protein-activated kinase; CHE, chelerythrine; ER, endoplasmic reticulum; ERK, extracellular signaling-regulated kinase; HNSCC, head and neck squamous cell carcinoma; IC50, a concentration that causes 50% inhibition; JNK, Jun N-terminal kinase; MAPK, mitogen-activated protein kinase; MMP, mitochondrial membrane potential; NSCLC, non–small cell lung cancer; PARP, poly-ADP-ribose polymerase; PKC, protein kinase C; ROS, reactive oxygen species; TNBC, triple-negative breast cancer; RCC, renal cell carcinoma.
